# Understanding the importance of quality control and quality assurance in preclinical PET/CT imaging

**DOI:** 10.1186/s40658-022-00503-w

**Published:** 2022-10-31

**Authors:** Wendy A. McDougald, Julia G. Mannheim

**Affiliations:** 1 BHF-Centre for Cardiovascular Science, College of Medicine and Veterinary Medicine, University of Edinburgh, Edinburgh, UK; 2 Edinburgh Preclinical Imaging (EPI), Edinburgh Imaging, University of Edinburgh, Edinburgh, UK; 3 Department of Preclinical Imaging and Radiopharmacy, Werner Siemens Imaging Center, Eberhard-Karls University Tübingen, Tübingen, Germany; 4 Cluster of Excellence iFIT (EXC 2180) “Image Guided and Functionally Instructed Tumor Therapies”, University of Tuebingen, Tübingen, Germany; 5 Centre for Cardiovascular Science, Queen’s Medical Research Institute, University of Edinburgh, Edinburgh BioQuarter, 47 Little France Crescent, Edinburgh, EH16 4TJ UK

**Keywords:** Preclinical PET/CT, Calibration, Quality control/assurance, Experimental design

## Abstract

The fundamental principle of experimental design is to ensure efficiency and efficacy of the performed experiments. Therefore, it behoves the researcher to gain knowledge of the technological equipment to be used. This should include an understanding of the instrument quality control and assurance requirements to avoid inadequate or spurious results due to instrumentation bias whilst improving reproducibility. Here, the important role of preclinical positron emission tomography/computed tomography and the scanner's required quality control and assurance is presented along with the suggested guidelines for quality control and assurance. There are a multitude of factors impeding the continuity and reproducibility of preclinical research data within a single laboratory as well as across laboratories. A more robust experimental design incorporating validation or accreditation of the scanner performance can reduce inconsistencies. Moreover, the well-being and welfare of the laboratory animals being imaged is prime justification for refining experimental designs to include verification of instrumentation quality control and assurance. Suboptimal scanner performance is not consistent with the 3R principle (Replacement, Reduction, and Refinement) and potentially subjects animals to unnecessary harm. Thus, quality assurance and control should be of paramount interest to any scientist conducting animal studies. For this reason, through this work, we intend to raise the awareness of researchers using PET/CT regarding quality control/quality assurance (QC/QA) guidelines and instil the importance of confirming that these are routinely followed. We introduce a basic understanding of the PET/CT scanner, present the purpose of QC/QA as well as provide evidence of imaging data biases caused by lack of QC/QA. This is shown through a review of the literature, QC/QA accepted standard protocols and our research. We also want to encourage researchers to have discussions with the PET/CT facilities manager and/or technicians to develop the optimal designed PET/CT experiment for obtaining their scientific objective. Additionally, this work provides an easy gateway to multiple resources not only for PET/CT knowledge but for guidelines and assistance in preclinical experimental design to enhance scientific integrity of the data and ensure animal welfare.

## Introduction

In preclinical research positron emission tomography/computed tomography (PET/CT) is a well-established widely used technique for in vivo imaging of small laboratory animals [1, 2]. PET coupled with CT (X-ray) provides researchers with a tool for gaining in-depth understanding of disease development, progression, drug therapy and radiotracer development. PET/CT supports investigation and evaluation of underlying biological mechanisms and physiological processes in healthy, as well as in diseased subjects/models.

In order to acquire a PET image, the laboratory animal is injected with a biologically active compound labelled with a positron emitting radioisotope (also referred to as radiotracer) prior to imaging. Radioisotopes are labelled to small organic molecules, antibodies and/or peptides. Generally speaking, the most common and widely used radiotracer is 2-deoxy-2-[^18^F]fluoro-D-glucose ([^18^F]FDG), a radiolabelled glucose analogue tracing the glucose consumption in vivo [3].

**Fig. 1:**
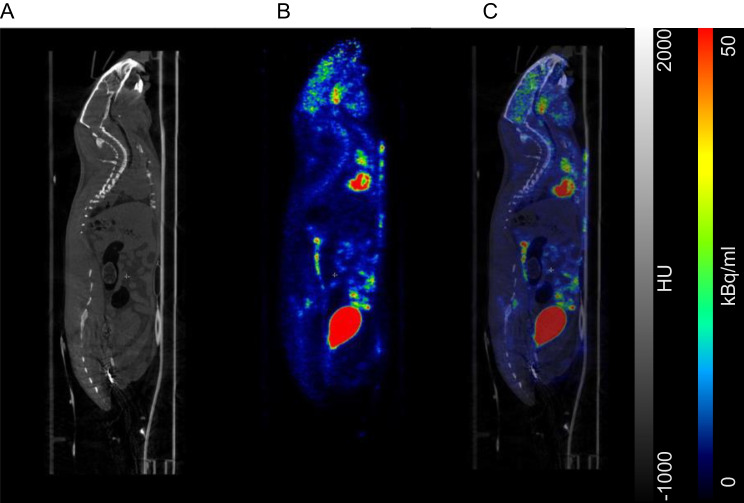
PET/CT sequence of images (CT, PET and PET/CT) of a rat with a myocardial infarct following injection of [^18^F]FDG administered via bolus injection into the tail vein. High [^18^F]FDG tracer uptake is seen in the heart as well as the discharge in the bladder. Panel **A** displays the CT (X-ray) anatomical information. Panel **B** shows the PET image (functional information). Panel **C** displays the PET/CT fused images. Scale shows CT Hounsfield units (HU) and PET tracer uptake in kBq/ml. McDougald W. PET/CT. In: Imaging Modalities for Biological and Preclinical Research: A Compendium, Volume 2. IOP Publishing; 2021:III.2.a-1-III.2.a-12. https://doi.org/10.1088/978-0-7503-3747-2ch18

The choice of radiotracer is dependent on the research question and the experimental design. Using target-specific positron emitting radiotracers in conjunction with X-ray, PET/CT allows for the acquisition of anatomical and functional information in one bed position (see Fig. 1). PET provides quantitative biological functional data information, whilst CT provides the anatomical information [4]. Examples for PET imaging include assessment of neurological diseases, cardiovascular disease, oncology, therapeutic drug discovery or radiotracer development.

Unfortunately, the use of imaging devices for research has the potential to introduce instrumentation biases, thus, possibly generating inadequate, confounding results and hence impacting the value of laboratory animals. Quoting directly from Russell and Burch, *The Principles of Humane Experimental Technique* (1959), "Inadequate research is wasted research, and cannot be tolerated indefinitely" [5]. Russell and Burch used that statement as a reference to experimental efficiency and efficacy—the capacity of the experiments to provide the required information [5]. Russell's and Burch's statement and foundational principles regarding the use of animals in research still hold true today. The National Centre for the Replacement, Refinement and Reduction of Animals in Research (NC3Rs, https://www.nc3rs.org.uk/) is a prime example of the dedication and applications of these principles. Their support and guidance provided to the preclinical research community for the replacement, refinement, and reduction (3Rs) of animals in research facilitates upholding these principles. However, the question is how this relates to instrumentation bias, QC/QA and the impact on results, specifically in preclinical PET/CT.

It is the responsibility of the researcher to ensure appropriate experimental design. At the very basic level, good scientific practice includes a well-designed experiment sufficient to achieve the scientific objective [6, 7]. Today, multiple resources are available which provide insight, guidance and identify the fundamentals for creating a well-designed experiment. For instance, the Animal Research: Reporting of In Vivo Experiments (ARRIVE) guidelines define a checklist of ten essential requirements for rigorous and transparent reporting [8]. This checklist also serves as a guideline for improved experimental design and statistical analysis. Accompanying the ARRIVE guidelines is documentation (Explanation and Elaboration) detailing the rationale for the checklist [8]. Examples of other resources can be found in clinical research literature outlining "acceptable practices", which address research validity, reliability, reporting, reproducibility, and clinical applications [9–11]. Henderson et al*.* (2013) investigated failed translational trials (preclinical to clinical medical interventions) and conducted a systematic review of preclinical research guidelines and recommendations [12]. From this study, Henderson et al*.* (2013) established two checklists: (1) design and evaluation of preclinical studies to support translation to clinical trials (Studies of Translation, Ethics and Medicine (STREAM)) and (2) reporting preclinical findings (Preferred Reporting Items for Systematic Reviews and Meta-Analyses (PRISMA)) [12]. Similar to ARRIVE, Henderson et al*.* (2013), Stout et al*.* (2013), Vanhove et al. (2105), Mannheim et al*.* (2017), Han et al*.* (2018) and others put forward guidelines directly relevant to designing and reporting animal studies which included: procedures (blood sampling, diet, circadian rhythm), number of animals required, housing, handling, randomization, blinding and more [8, 12–17].

For preclinical researchers using PET/CT systems the experimental design should also include: (1) selecting the proper imaging radiotracer for PET and/or contrast agent for CT, (2) how the administration of any therapeutics, anaesthesia, radiotracers and/or other agents is to be carried out, (3) the appropriate animal model and (4) the PET/CT system's performance, imaging protocols and reconstruction methods (both PET and CT) [13, 14]. Moreover, in Baker's *Nature* (2016) survey 90% of the scientists surveyed requested "more robust experimental design" [18]. This directly leads back to expanding the knowledge, education, and responsibility of researchers using PET/CT to include a basic understanding of the instrumentation and its performance records/evaluations. Therefore, and noted as the fourth requirement above for PET/CT users, the appropriate imaging protocols to be used should be discussed with the PET/CT supervisor or director during experimental design along with the verification of scanner QC/QA.

In sharp contrast to clinical study design, the majority of preclinical studies are conducted as a single-laboratory study and not as a multicentre study as in the clinical setting. This is partially due to the fact that for preclinical studies the required animal number for statistical power/significance can be achieved within a single institution. Whereas, in order to reach a sufficient patient cohort for statistical significances most clinical studies need to be performed in a multicentre approach. However, a distinct advantage of performing preclinical studies in a multicentre approach might be the improvement in the scientific integrity of data. For this, proper QC/QA guidelines need to be in place to ensure the comparability of data on a multicentre level. Nevertheless, given the fact that preclinical molecular imaging is still in the early stages of setting up standardized multicentre protocols, referencing already implemented clinical PET/CT protocols and QC/QA regulations can assist in developing preclinical PET/CT standards.

Clinical PET/CT standardized protocols also aid the preclinical scientific community with experimental design information. First and foremost, a PET/CT (clinical or preclinical) image is critically dependent on scanner's performance. For PET this means the scanner's ability to detect gamma rays emitted via positron–electron annihilation from the injected radiotracers [19]. Neglecting QC/QA causes the scanner to eventually inadequately detect, count and collect the emitted gamma rays. Thereby, effecting imaging quality and most importantly the empirical quantitative data analysis [14, 15, 20]. For CT, it is critical that the X-ray tube is emitting the correct amount of ionizing radiation as well as detecting. Poor scanner performance might generate instrumentation biases leading to spurious results. Straightforwardly put, instrumentation bias is defined as deficiencies in the calibration or maintenance of measurement instruments, causing systematic deviations from true values [21].

Acquiring inadequate, unrepeatable, and unreproducible imaging data should not be tolerated. Therefore, this paper focuses on current literature regarding preclinical PET/CT scanner QC/QA techniques, technically outlining PET/CT QC/QA procedures, and standardization whilst noting references for detailed PET/CT QC/QA guidelines. Furthermore, quantitative image analysis discrepancies and inaccuracies are presented. It is important to recognize that a visual image quality "check" will not always uncover failed detector blocks, thus, leading to inaccurate image data analysis. Therefore, providing researchers with the knowledge of PET/CT scanner's vital performance requirements allows for the correct questions to be asked when designing experiments and caring out the quantitative image analysis.

## Preclinical PET/CT scanner quality control

**Fig. 2:**
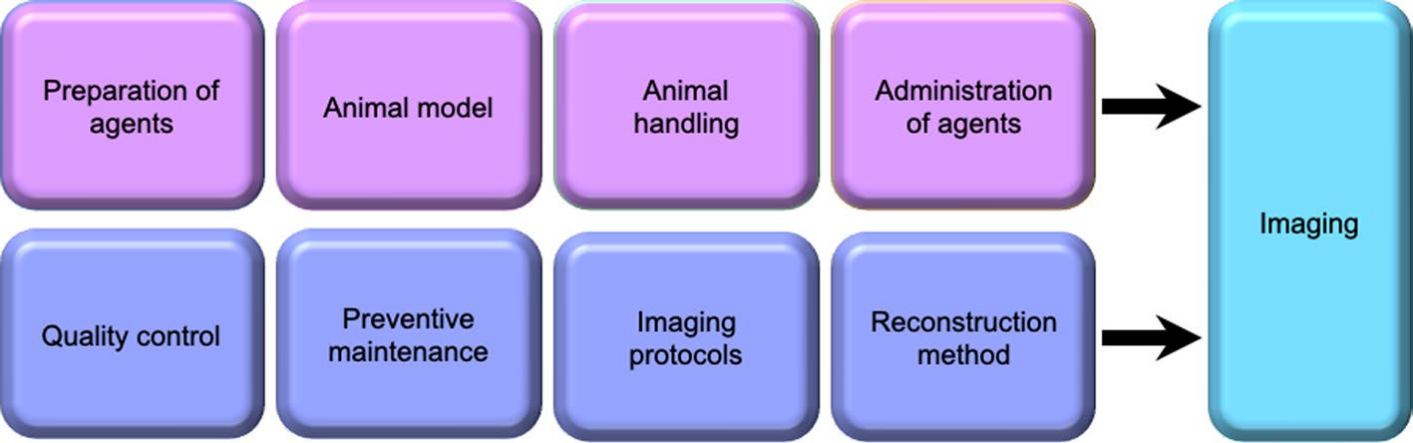
Schematic representing factors that impact acquired imaging data sets. Animal influencing factors are shown on the top, whereas QC/QA and protocols (acquisition and reconstruction) are on the bottom. Modified from Vanhove et al. (2015) [14]

Over the last 10 years several excellent publications addressing the importance and the many facets of preclinical standardization have been produced [6, 12–15, 20, 22–26]. Multiple published reports, reviews and analysis comparisons of preclinical studies highlight the problem of the irreproducibility of preclinical data results [27–30]. The publications all have the common thread of targeting preclinical efficacy, reliability, and reproducibility not only for the validity of research and the welfare of laboratory animals but also for quality, robustness, and relevance as well as translation capabilities to clinical studies. Figure 2, a simplistic outline, demonstrates the multiple factors influencing imaging data sets [14]. During the initial steps of experimental designing each of these factors need to be discussed and fully encompassed into the study. However, the significance of scanner QC is rarely discussed and yet understood amongst the scientific community to be a significant concern. Establishing routine ongoing QC measurements, visual and quantitative analysis, and preventative maintenance, verifies that the system is performing optimally within preset ranges. This section focuses on the elucidation of required QC techniques both for PET and CT.

### PET

The standardized clinical PET scanner requirements and guidelines [31–34] established the foundation for the preclinical guidelines. Now, primary preclinical scanner performance requirements have been set by the National Electrical Manufacturers Association (NEMA). Recognized world-wide, NEMA is a forum for the standardization of electrical equipment, including medical imaging scanners. In collaboration with members of the Society of Nuclear Medicine and PET scanner manufacturers, NEMA published its first clinical PET guidelines for standardized performance measurements in 1994 [35, 36]. By 1996 these guidelines were modified and adopted specifically for the newly developed preclinical small animal PET imaging systems. Today, version NU 4-2008 describes the standard performance evaluations every preclinical PET scanner should undergo [37]. However, more recently Hallen et al*.* (2020) critically discussed the NEMA NU 4-2008 standards, noting flaws and outlining suggestions for future discussion and potential improved procedures [38].

As mentioned in the introduction, PET scanners detect, count, and collect emitted gamma rays. Hence, NEMA scanner performance measurements will test—among other things—scanner sensitivity, scatter corrections, spatial resolution, and image quality. Sensitivity relates to the scanner's ability to detect the gamma rays. It is defined as the rate of counts per second of true events, where true events are gamma rays detected in coincidence. Not all events are "true", thus, the system will also detect scattered and random photons. This produces false and mispositioned detected events and needs corrections (scatter and random corrections). Spatial resolution is determined by the scanner's ability to distinguish separate points after reconstruction of the image. The image quality test relates to the uniformity of the image, the resolution, and the accuracy of data corrections.

All these parameters and characteristics will impact quantitative and visual analysis if not performing at an acceptable level. Thus, applying and demonstrating compliance with NEMA testing requirements provides an acceptable system performance level. It also generates "typical" imaging conditions expected for maintaining the system integrity and comparison of different systems given the complexities between them. Unfortunately, but also given the extent of procedures, NEMA testing is typically only performed upon installation of a scanner and not routinely. Greater details of the preclinical NEMA protocol can be found via reference [37].

**Fig. 3:**
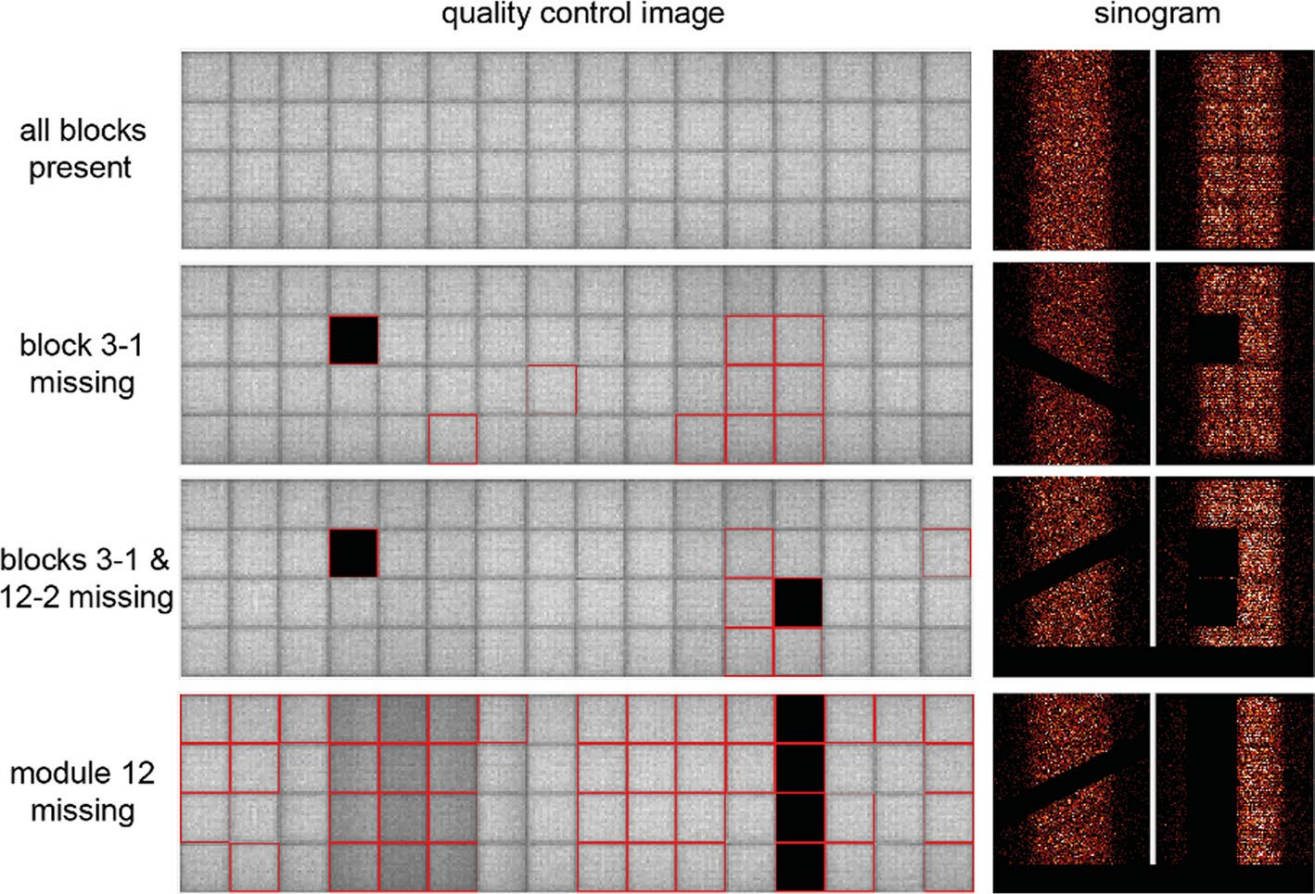
Exemplary study demonstrating the impact of missing detector blocks on the daily quality control and the sinogram data

One of the paramount advantages of a regularly performed quality control is, besides ensuring stability and reliability of the acquired data, that possible hardware problems can be detected at an early stage. This is especially important as most of these hardware problems might not be visible or detectable in the reconstructed research data. For instance, Fig. 3 displays an exemplary study performed in-house using a homogeneous ^68^Ge phantom investigating the impact of one or more missing detector blocks on the daily quality control and the sinograms. The ^68^Ge phantom was scanned for the respective four cases (all blocks present, one block missing, two blocks missing, four blocks missing) to determine the impact qualitatively and quantitatively on phantom data. The sinogram data (Fig. 3) clearly show the missing blocks, quickly indicating that the scanner is malfunctioning. Here the importance of a proper implemented and regularly performed quality control is demonstrated, as missing or malfunctioning blocks would directly be detected before performing the actual animal image acquisitions.

**Fig. 4:**
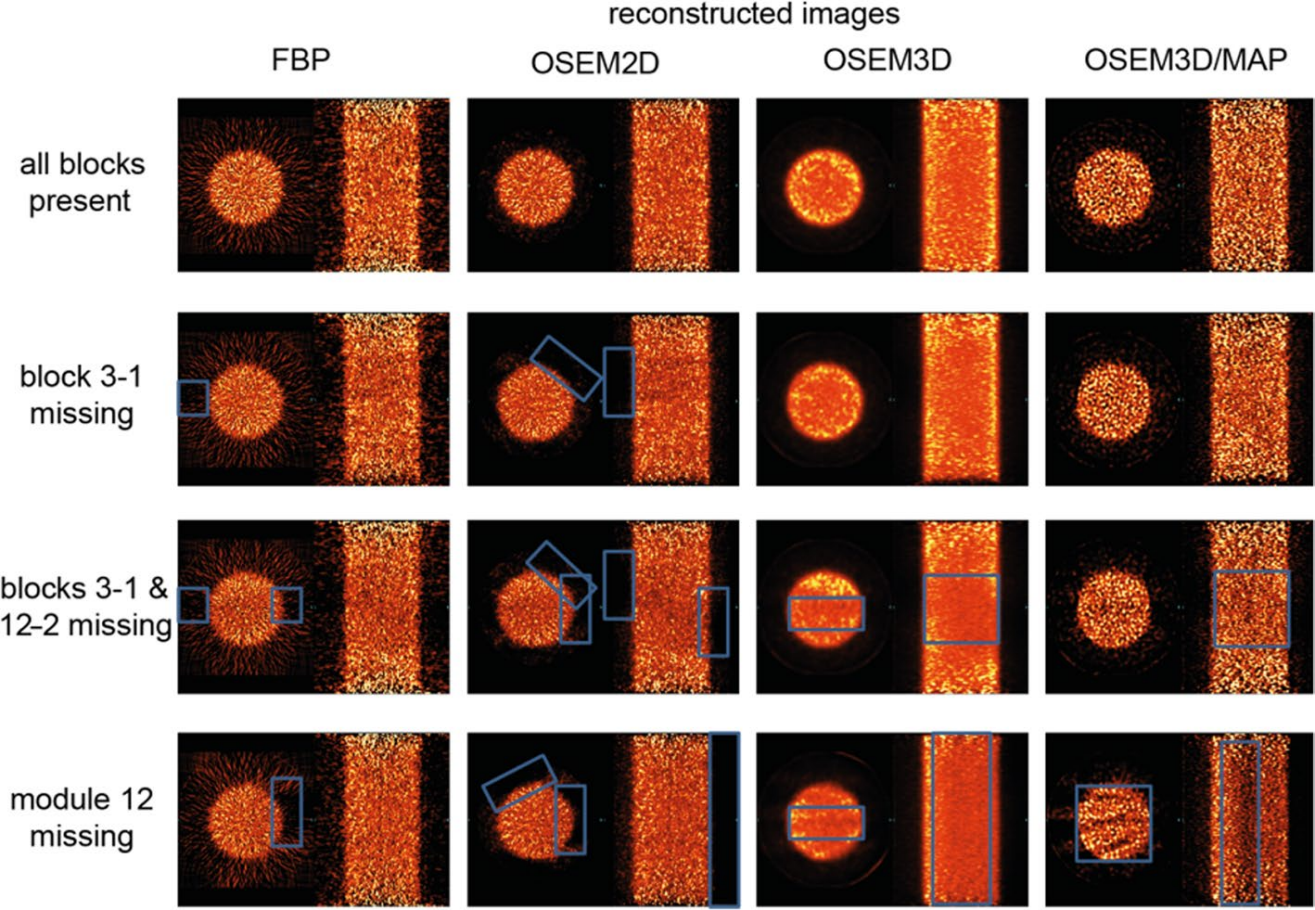
Reconstructed images of a homogeneous phantom reconstructed with 2D filtered backprojection (FBP), ordered subset expectation maximization (OSEM) in 2D and 3D and a combination of OSEM3D with maximum a posteriori (OSEM3D/MAP)

However, for the reconstructed images shown in Fig. 4, the impact is more difficult to detect qualitatively. Especially with just one block missing, the impact is only hardly detectable as the phantom still shows a homogenous uptake pattern. Generally speaking, most researchers would not recognize the malfunction of one detector block based on the reconstructed images only, and unfortunately, the sinogram data are typically not reviewed at all. When two blocks or an entire module consisting in this case of four detector blocks are missing or malfunctioning, the impact on the reconstructed images becomes more obvious. Interestingly, the impact of the missing blocks visible in the reconstructed images is dependent on the different reconstruction algorithms. Especially for the 2D algorithms it appears that the qualitative impact on the data is more visible compared to 3D algorithms. Please note that this might of course be dependent on the used reconstruction parameters (e.g. number of iterations or subsets, filters).

**Fig. 5:**
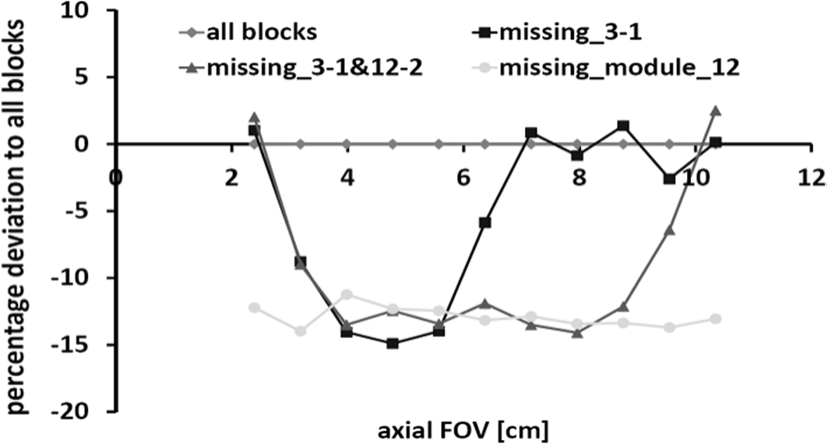
Percentage deviation along the axial field of FOV for the three investigated cases (one block missing, two blocks missing, and four blocks missing) based on when all blocks are present, revealing substantial deviations

The quantitative impact of missing detector blocks on standard uptake values (SUVs) along the axial field of view (FOV) is displayed in Fig. 5. The deviation of SUVs was calculated compared to when all blocks were functioning. If ‘only’ one block is missing deviations of up to 15% are detected depending on the position of this block along the FOV (analysed for OSEM2D reconstructed images). When two blocks are missing, a larger fraction of the axial FOV is affected with deviations of up to 15%. Finally, when an entire module consisting of four detector blocks is missing, deviations of up to 15% along the entire axial FOV are detected. This clearly demonstrates the significant impact of missing and malfunctioning detectors and hence the importance of QC/QA. If such a malfunction in an animal image acquisition is not recognized, the researcher could interpret these deviations as a biological relevant change representing the change in the underlying research application.

**Fig. 6:**
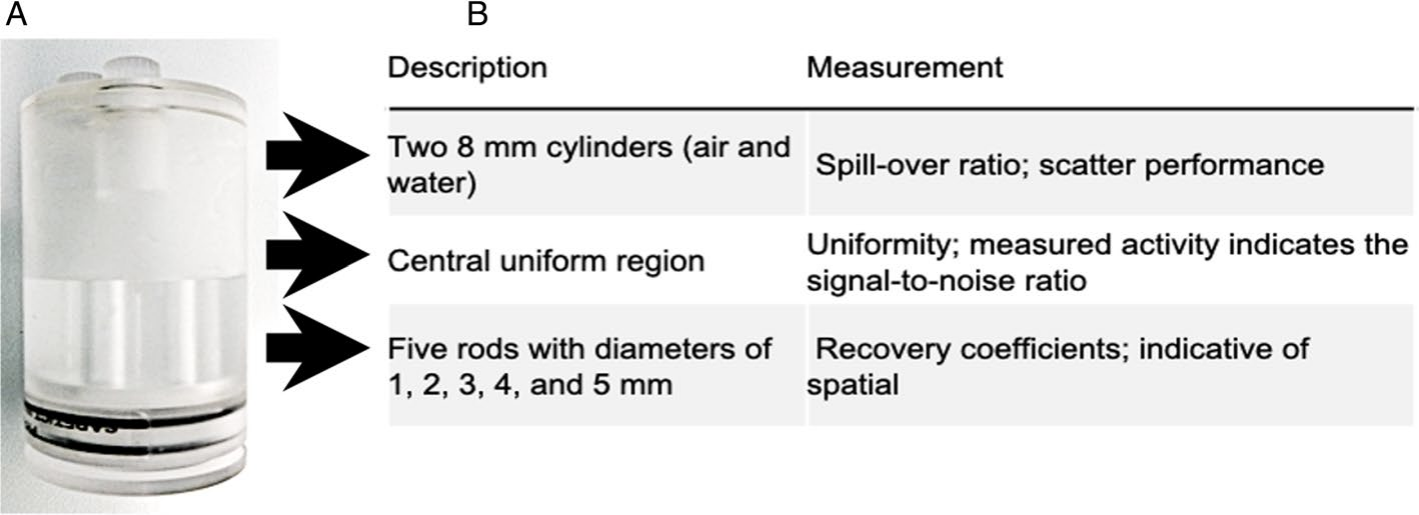
Three-chambered PET preclinical image quality phantom (IQ) (6.3 × 3.5 cm), used for QC/QA. Panel **A** displays an image of the IQ phantom. Panel **B** outlines the individual chambers/sections of the phantom as well as provides brief explanation of the evaluation parameters acquired

System QC testing should be carried out frequently for optimal performance and as warranted by daily observations of the scanner and room environment (i.e. changes in room temperature/humidity) stability [24, 39]. A variety of PET phantoms are available to perform QC. A basic and easy-to-use phantom is a homogeneous ^68^Ge cylinder or a sealed cubic ^22^Na source, which can be used for daily quality control testing and to determine the long-term stability to ensure reproducibility. The most widely used phantom that can be filled, also the recommended NEMA phantom for evaluation purposes, is the commercially available Image Quality (IQ) PET phantom (Fig. 6). The preclinical IQ phantom, a 3.5 cm diameter cylinder, can be filled with a measured activity concentration of water mixed with a radionuclide, e.g. ^18^F, prior to imaging. Multiple parameters can be evaluated, such as recovery coefficients of different rod sizes, uniformity and spillover ratios in water and air compartments to determine the performance of the tested system, as well as to investigate long-term stability.

The European Association of Nuclear Medicine (EANM), physics group on Nuclear Medicine Instrumentation Quality Control, has set out a routine testing schedule guideline for preclinical scanners (Table 1) [24]. As the largest organization in Europe committed to nuclear medicine, EANM has a long history of focusing on the improvement in nuclear imaging techniques and routines, the education and exchange of knowledge in nuclear medicine.

**Table 1:** Routine PET quality control testing; daily, weekly and quarterly

Test	Purpose	Frequency	Comments
Physical inspection	General check	Daily	Checking for mechanical or other defects that can lead to system failures
Background count rate	Detection of excessive electronic noise	Daily	Comparison to baseline/reference value
Detector check	Functional check of detectors/blocks	Daily	Call service if a detector block failed
Energy resolution	Verification of the systems energy resolution by summing the energy spectra of the individual detectors/blocks	Quarterly	Repeat calibration if the energy resolution is greater than the acceptance test measured energy resolution; photopeak verification of each detector/block to check if it correctly matches the 511 keV value
Pixel identification	Verification of correctly assigned pixels to the corresponding scintillating element	Quarterly	If mismatch is detected, repeat pixel identification
Sensitivity-quantitative	Evaluation of the systems sensitivity	Quarterly	Follow test procedures as described in the NU 4-2008 performance manual
Spatial resolution	Evaluation of the systems spatial resolution	Quarterly	Follow test procedures as described in the NU 4-2008 performance manual
Image quality (Uniformity, recovery coefficients, spillover ratios)	Determine uniformity, recovery coefficients, spillover ratios	Quarterly	Follow test procedures as described in the NU 4-2008 performance manual
Attenuation and scatter correction	Determine effectiveness of attenuation and scatter correction	Quarterly	Follow test procedures as described in the NU 4-2008 performance manual

Modified, EANM Physics Group on Nuclear Medicine Instrumentation Quality Control 2010 [24]. NEMA document NU 4-2008 [37]

By implementing regular and routine QC procedures, scanner consistency in performance results can be assured and potential drifts monitored. Quarterly NEMA testing requires using the IQ phantom and an ^18^F solution. Daily and weekly tests are best performed using a long-lived sealed radionuclide such as ^22^Na or ^68^Ge encapsulated in a polypropylene polymer material (cube or cylinder) [20].

### CT

To date, CT QC has mainly been developed for clinical systems, which use different tube voltages (kVp) and currents (mA) from that of preclinical CT. However, the fundamental QC concepts, applications and measurements set out clinically by governing bodies and research sites/institutions such as the International Atomic Energy Agency (IAEA), the American College of Radiology (ACR), EANM or the International Electrotechnical Commission (IEC) can be applied preclinically as well [40–43].

Prior to hybrid CT systems (PET/CT, SPECT/CT, optical/CT), preclinical CT was mainly used as a stand-alone scanner in research for in vitro and in vivo imaging. Though it is still used in this regard today, multimodality systems that acquire functional data (PET, SPECT or optical) sequentially with CT are more common, hence increasing the importance of implementing QC routines. Regardless of the governing body or research facility, the three main QC testing criteria agreed upon are:

- air and water Hounsfield unit (HU) assessment (also known as CT numbers)
- visual artefact evaluations and
- CT alignment with PET.

All these three parameters can be determined with a CT quality phantom filled with water.

**Fig. 7:**
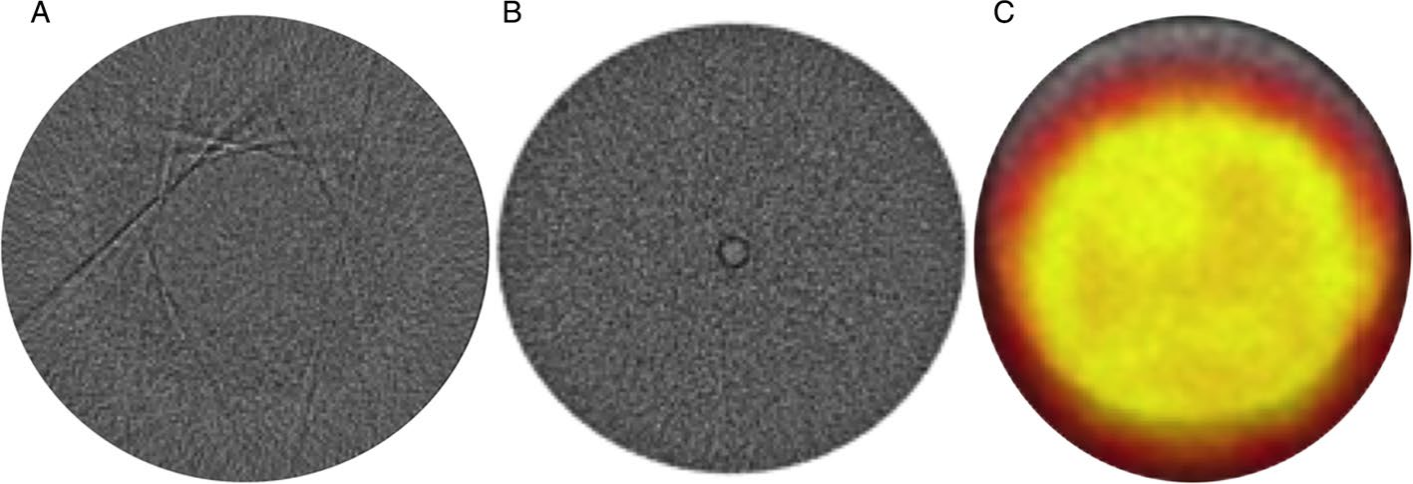
Images displaying common examples of artefacts that would be seen by visual QC inspection of CT images. Panel **A** displays an example of CT streaking artefacts. The cause of such streaking artefacts is most of the times due to mechanical failure or imperfections (poor detector sampling and failure). Panel **B** shows an example of a CT ring artefact mostly caused by a CT centre of rotation error. Panel **C** is the axial view of a PET/CT phantom showing the misalignment between the PET (colour) and the CT (light grey arc). This is due to scanner hardware being misaligned. McDougald W. PET/CT. In: Imaging Modalities for Biological and Preclinical Research: A Compendium, Volume 2. IOP Publishing; 2021:III.2.a-1-III.2.a-12. https://doi.org/10.1088/978-0-7503-3747-2ch18

QC will disclose incorrect HUs which are mainly due to incorrect calibration, software or hardware malfunctions. Visual artefacts can easily be seen during the daily QC in the water chamber of the CT Quality Control phantom. Misalignment generates a mismatch between CT and the functional imaging data sets. This should be caught and corrected anytime seen, although, regular performed QC will reduce the possibility of misalignment. Examples of these artefacts and errors are displayed in Fig. 7. As stated, poor scanner performance on any of these levels will impact quantitative and visual analysis.

Furthermore, inaccurate CT image data might impact PET image quality and quantitative analysis as PET/CT scanners depend on the anatomical information from the CT data (HU values) for generating attenuation corrections in PET. Inaccurate HU, whether misalignment or miscalibration, lead to instrumentation biases potentially causing an underestimation or overestimation of the radiotracer activity in the PET data.

The measurement in X-ray is essentially the spatial distribution of the linear attenuation coefficient (basically how the X-rays travel). This measurement, though dependent on energy and medium, is assigned a Hounsfield unit (HU) value, relative to the attenuation of water. Therefore, different tissues, organs are scaled accordingly to generated HU values based on the following formula.

1 $${\text{CT }}\,{\text{value}}\, \left( {{\text{HU}}} \right) = 1000\frac{{\left( {\mu - \mu_{{{\text{water}}}} } \right)}}{{(\mu_{{{\text{water}}}} - {\upmu }_{{{\text{air}}}} )}}$$

with *μ* being the linear attenuation coefficients [44–46].

**Fig. 8:**
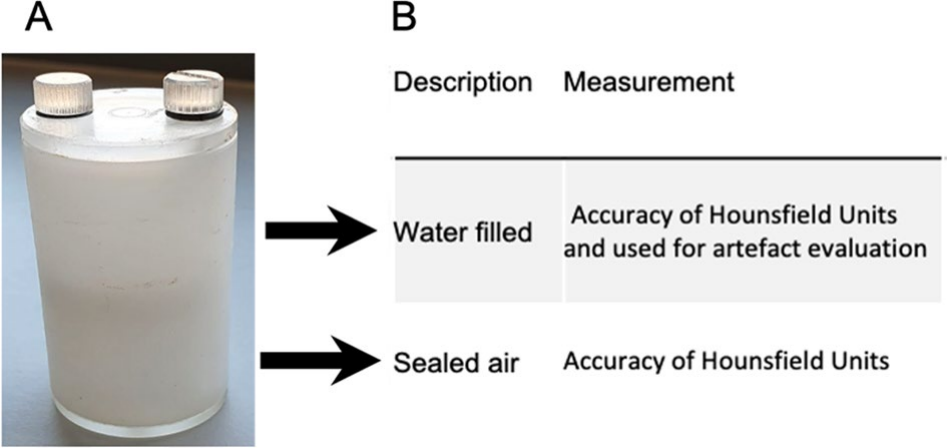
Two-chambered CT image quality control phantom (QC) (6.5 × 3.5 cm), used for QC/QA. Panel **A** displays an image of the QC phantom. Panel **B** outlines the individual chambers/sections of the phantom as well as provides brief explanation of the evaluation parameters acquired

The same reasoning and fundamental concepts as in PET hold true for CT; system QC testing should be carried out frequently for optimal performance and as warranted by daily observations of the scanner [24, 42, 47]. Figure 8 displays the basic commercially available preclinical QC CT phantom, 3.5 cm diameter cylinder, to be used in testing. The top chamber of the QC phantom will be filled with water at the time of imaging. Table 2 outlines a routine testing schedule guideline set out by the EANM, group on Nuclear Medicine Instrumentation Quality Control, for CT scanners [24].

**Table 2:** Routine X-ray CT quality control testing; daily, monthly and quarterly/annually

Test	Purpose	Frequency	Comments
CT	Daily procedures	Daily	Follow manufacturer’s recommendations (e.g. tube warm-up)
CT/PET	Determine co-registration vector of PET and CT field of view	Daily	Check PET and CT image alignment
CT HUs	Determine CT HUs accuracy	Daily	CT HUs accuracy: water and air and standard deviation (noise)
CT artefacts	Evaluation of artefacts	Daily	Visual inspection of the water image slices from the CT QC phantom
CT performance	Check X-ray performance and radiation exposure	Based on national legislation	Follow national legislation guidelines
CT grey level performance	Check scanner acquisition display monitors	Monthly	Check quality of monitors grey scale for image display (see also Society of Motion Picture and Television Engineers (SMPTE) test) [48, 49]

Modified, EANM Physics Group on Nuclear Medicine Instrumentation Quality Control 2010 [24, 50]

Beside the basic preclinical CT QC phantom used for daily and quarterly/annual testing multiple other CT phantoms are available that, e.g. include a tissue equivalent material (TEM) (rods of different densities representing bone, lung and soft tissue) or spatial resolution bars (bar patterns from 5 to 150 μm lines). Though these are not used for daily testing, the TEM phantom should be used regularly for HU evaluation and validation of different densities.

It should be noted that all systems have the necessary internal software installed in order to facilitate QC testing. Therefore, establishing daily, weekly, and quarterly QC routines is feasible and attainable. In addition, the implementation of regular and routine QC procedures assures consistent scanner performance results and allows for trends to be monitored. Even if a research institute has a vendor service contract, in-house QC still needs to be established and carried out regularly. Established QC routines should not supersede or replace manufacturer recommended QC and preventive maintenance [14, 19]. Greater details of CT tests outlined by EANM and ACR can be found via reference, 50 and 51, respectively [50, 51].

## Preclinical PET/CT scanner quality assurance

Image quality and the empirical quantitative data is dependent not only on scanner daily characteristics performance but also on the calibration and maintenance of the scanner. In the context of the PET/CT scanners, QA relates to and ensures the scanner is operating at the acceptable levels established by the scientific community such as NEMA standards [37, 51]. Therefore, the following two sections briefly cover necessary combined PET/CT scanner calibrations to enable reproducible and reliable sequential acquisitions.

Preclinical calibrations, maintenance and annual testing are similar to the procedures and protocols used on clinical PET/CT scanners, though not carried out as often. Additionally, preclinical calibration procedures tend to be less rigorous and extensive especially in CT calibration and quality assurance. Calibration tests done by the vendor engineer or in-house engineer set the baseline for the regular quality control performance testing. The baseline values are used for the scanner corrections factors applied to the image data. Currently, there is no requirement for scanner accreditation at preclinical research sites or institutes. For this reason, it behoves the researchers to validate proper and regular scanner QA is conducted.

### PET calibration and quality assurance

Maintenance and scanner calibrations should be done regularly (e.g. monthly, quarterly, semi-annually, or as suggested by the manufacturer) [20]. All manufacturers provide diagnostic software for QC testing, calibration or quality assurance testing, however, the software features might vary greatly from manufacturer to manufacturer.

In general, the first step in calibrating a PET scanner is to use the provided manufacturers system diagnostics software (if available). These software programs can allow for running analytical evaluations on the detector system (scintillations crystals, detectors, and read-out electronics). The resulting software assessments can provide information on the detector gains and energy (i.e. photomultiplier tube outputs), crystal positions/read-out (crystal maps) and coincidence timing evaluation. Corrections, updates of gains, energy, position, and timing are performed during detector setup and potential hardware changes (e.g. replacements of detectors or circuit boards) can be determined from this information. The detector setup is the base for a stable performing PET system. In addition, blank scans (nothing in the scanner's field of view) using 2D and 3D mode can be acquired to evaluate sinograms (collected image projections). Along with these diagnostic evaluations, visual inspections of internal components and filter cleaning need to be carried out.

Once any modification or corrections are finalized, a normalization scan is usually acquired to normalize the detectors (e.g. using a ^68^Ge cylindrical source). The time of the normalization scans will slightly vary based on the used activity and on the manufacturer's recommendation and is usually in a range of a couple of hours [20].

Secondly, following the normalization, a calibration of the scanner is performed to convert acquired counts in activity concentration units by using a cylindrical water phantom filled with a defined amount of fluorine-18 (^18^F) or the default isotope in use [20]. Generally, the scan times are 20 min but may vary depending on the system and on the manufacturer's recommendation. Importantly, the calibration data should be reconstructed using the same reconstruction algorithm and the same corrections applied (e.g. attenuation, scatter) as used for the actual studies afterwards. Acquisition of the phantom provides the scanner's global activity correction factor, which also corrects for internal scanner variations. The calibration of a scanner also sets a "new" baseline for the expected scanner performance.

### CT calibration and quality assurance

As with PET, CT maintenance and scanner calibrations are performed regularly (e.g. monthly, quarterly, semi-annually, or as suggested by the manufacturer) by using the provided diagnostic software for QC testing, calibration or quality assurance testing [20].

As a first step, visual inspections of internal components, and filter cleaning are carried out. Second, using the manufacturer-specific diagnostics software along with the CT QA performance phantom placed inside the bore, assessment of the X-ray tube and detectors is carried out. A CT QA performance phantom contains several separate sections each designed to test a specific performance. The phantom is mainly composed of acrylic with sections consisting of different size lines or strips, circles, and an area with different density rods. This phantom allows for the testing of CT spatial resolution (high and low contrast scale) uniformity noise and slice thickness. Using the manufacturer-specific acquisition software, a diagnostic CT should also be carried out to determine CT numbers (HUs) for each tube voltage.

### PET/CT annual testing

Annual testing provides an additional level of assessing scanner consistency and stability in performance. The current gold standard guidelines on annual testing for PET can be found in the NEMA NU 4-2008 preclinical evaluation protocols [37] and under the American College of Radiology (ACR) for clinical PET and CT [51].

Clinically and preclinically, PET annual testing mainly focuses on evaluating scanner performance measurements for sensitivity and spatial resolution in air and in scattered medium. Annual testing currently covers sensitivity (ability to detect/count), count rate, scatter fraction (scattered photons), coincidence timing window (detect true within correct coincidence time), count losses and random events, accuracy of corrections (attenuation and scatter), time of flight (if applicable) resolution, and co-registration accuracy with CT [37]. For preclinical scanners, this is accomplished using the PET IQ phantom filled with water and a measured specific amount of ^18^F (with areas for activity and no activity measurements) and a phantom designed as a line source filled with a relatively high activity of ^18^F [37].

The annual testing of CT focuses on measurements of the X-ray tube beam energies, ionizing radiation, absorbed dose and detector performance. Annual testing on CT also assists in monitoring correct absorbed doses, thus, potentially avoiding overexposure due to incorrect X-ray beam energies. This test uses a similar CT phantom to the one used in CT calibrations with an ion chamber and radiochromic film. This phantom consists of four sections to evaluate high contrast, uniformity/noise, low contrast, and alignment, with each section containing the necessary density rods and lines/circles. Similar to calibration, these parameters are measured along with the tube current (mAs) linearity and slice thickness. Annual CT testing also covers measuring the CT dose index using an ion chamber, for several tube voltages and/or tube current. The X-ray beam width is measured using the radiochromic film for each slice thickness using the routine CT protocols.

## Discussion

Suboptimal scanner performance does not provide researchers accurate, reliable, robust translational imaging data sets nor is it cost effective and in accordance with animal welfare. In fact, it is quite the opposite. Inadequate animal acquisitions due to scanner poor performance result in higher monetary costs and generate insufficient image data sets. Furthermore, animal welfare is a significant concern; inconclusive or unreliable experiments carry the cost of potentially causing unnecessary use and harm to animals or loss of life [14, 15, 20, 26, 52–54]. Based on the example of Fig. 1, in which a rat has undergone an operation to create a heart infarct: on the day of imaging the rat is injected with anaesthesia, a PET radiotracer and most likely a CT contrast agent. If only imaging once, euthanasia will probably occur after imaging. In the case of a longitudinal study the imaging process (injections and radiation) will be repeated multiple times. Next consider any rodent cohort sample size. If the scanner isn't correctly functioning (not detecting and collecting counts) all the quantitative analysis is inaccurate, questionable, and basically inconclusive. This study would have unnecessarily inflicted harm and possibly loss of life to the animals. This would be harm and loss without true scientific benefit due to instrumentation bias. Biases that could have been avoided if QC/QA had been routinely carried out and properly maintained [55].

Clinical research evaluating the impact on instrumentation bias (miscalibration) of PET/CT scanners found that patient quantitative SUV measurements across centres can vary up to 46% [31, 56–63]. Doot et al*.* (2012) considered what the impact of miscalibrations on patient sample size for Phase II clinical trials would be [64]. They set the parameters as a two-armed study measuring FDG SUVs in tumours with a true difference of 20 percentage points between the groups as well as an effect size of 0.2, randomized at 0.05 and 80% power. Given the shown variability of up to 46%, Doot et al*.* (2012) calculated the sample size required to achieve the scientific objective as calibration errors worsened from 10%, 20% to 40% on a multicentre level. Their findings revealed the sample size increased from 10, 39 to 156, respectively, in direct correlation with greater measurement errors [64]. In 2009 Scheuermann et al*.* evaluated clinical SUV data submitted for scanner accreditation within the ‘ACR Imaging Network’ (ACRIN). Out of 169 received scanner applications, 101 applications were reviewed and only 36% passed without any intervention needed, whereas 56% of the scanners required intervention and corrections before passing. 8% of the scanners failed to pass [65]. Recently, it has been shown that preclinical measurements do vary across multiple centres by as much as 44% [22, 23]. Therefore, applying similar metrics to preclinical: the rodent *n* sample size required increases exponentially with miscalibration and 56% of preclinical PET SUV measurements are potentially inaccurate and/or invalid. Not only is this detrimental to research outcomes but it violates the 3Rs principles. Furthermore, in 2015 Ioannidis et al*.* estimated that 85% of invested effort and resources in biomedical research were wasted due to a variety of diverse inefficiencies [66]. Clinical literature indicates relative calibration errors account for up to 50% variation on SUV quantification [31]. As a preclinical example, our exemplary study on the effects of missing PET detector blocks (Figs. 3, 4 and 5) revealed a significant impact on the quantitative outcome, thus, introducing large quantitative biases within an animal study if not recognized. This can cause the biological interpretation of the data to be inconclusive or in the worst case to be wrong, which obviously should be strictly avoided.

However, the good news is in 2017 Scheuermann et al*.* found that consistent scanner qualification/calibration process helps ensure scanner performance for the entirety of a clinical trial [67]. Though, actual failures of research studies (or components of) from instrumentation inefficiencies are rarely, if ever, reported. This does question the robustness, reproducibility, and validity of said studies as well as the original experimental design and the possibility of selective reporting.

In CT, besides image quality and quantitative analysis, an additional critical reason for regularly implemented QC/QA routines again lies within animal welfare. A key component of the CT performance testing and calibrating is to ensure the X-ray beam measurements are correct. It is well known that ionizing radiation (X-rays) causes cells and DNA damage. This damage potentially impacts the animals, the biological responses from the radiation effect and can therefore impact the research study [68–71]. Incidental ionizing radiation causing over exposure of any laboratory animal is not conducive to good scientific practices nor ethical. In a recent study, the ionizing radiation dose small laboratory animals were receiving during one routine CT image acquisition was measured across five preclinical research centres each with different PET/CT scanners. Firstly, this study noted that more than one scanner was plagued by calibration errors requiring intervention from the manufacturer [23]. Secondly, measured CT ionizing radiation doses absorbed by mice ranged from 11 to 216 mGy, and by rats the range was from 7 to 100 mGy [23]. It should be noted the higher doses (100—216 mGy) would be considered radiation therapy doses and currently doses greater than 60 mGy are shown to cause DNA damage [68, 69, 71].

Research repeatability and reproducibility remain the most important fundamental principle of the scientific method and distinguishes scientific evidence from mere anecdote [52]. Experimental design plays a critical role in research outcomes of validity and reproducibility, warranting greater rigor. Fortunately, discussions and literature continue to address various, multifactored issues regarding the validity of preclinical research [7, 18, 53, 66, 72–74]. Now the push for improved experimental design, reposting, education and imaging standardization of protocols and QC/QA is becoming stronger [8, 14, 15, 23]. For example, the European Society for Molecular Imaging (ESMI) has established a coalition of preclinical imaging researchers (study group “Standardization of Small Animal Imaging”) whose priority is to standardize preclinical imaging, which also includes scanner QC/QA and protocols (https://e-smi.eu/esmi-study-groups/standard/). In the USA, the Society of Nuclear Medicine and Molecular Imaging (SNMMI) preclinical research website now suggests nine topics for establishing imaging guidelines for the preclinical community (https://www.snmmi.org/Research/PreclinicalImagingLandingPagePT.aspx?ItemNumber=3343&navItemNumber=750). Two of those topics are the "development of standardized image format and data analysis". Both the ESMI and SNMMI recent initiatives hold promise for global preclinical imaging QC/QA standardization requirements.

To summarize, the fundamental tenets for PET/CT QC/QA are:

1. QC must be performed on a regular, periodic basis. It is prudent to carry out at a minimum on the days when imaging. QC to test for reliable data will lower impact on animal welfare.
2. Prompt interpretation of measured QC/QA results is necessary for early recognition and remedy when systems produce inadequate results.
3. Diligent bookkeeping, record keeping of scanner performance is a vital component of QC/QA. Recorded results allow for quicker troubleshooting, validation of scanner performance and recognition of changes or malfunctions. Comparing current QC/QA results to past results will reveal changes and/or any degradation/drift.

Quality assurance ensures the scanner is operating at an optimal level and quality control maintains this performance. Parameters and recommendations of testing acceptance (pass or fail) are defined by NEMA, IEC, ACR, respectively [37, 43, 51].

## Conclusion

Embracing the principles of experimental efficiency and efficacy requires researchers to maximize their knowledge on the aspects and impacts of the imaging techniques or tools to be used. This includes understanding required scanner QC/QA and ensuring it is routinely carried out. Maintaining QC/QA will reduce scanner failure as well as allow for errors to be identified and corrections made prior to putting animals through the imaging process. Therefore, especially in longitudinal studies, any potential unnecessary harm or loss of life is reduced. Research, clinically and preclinically, has validated the serious impact instrumentation bias has on quantitative measurements.

It is estimated that upwards to 108,000 rodents have been imaged using PET/CT over the last five years (ISI Web Science search PET/CT rat and/or mouse). The study by Scheuermann et al. evaluated that 56% of clinical scanners did not pass the ACRIN review without intervention [65]. These interventions included a number of reasons, from relatively easy-to-fix incorrect dicom information to more severe miscalibrations. However, we want to point out here, that also relatively straightforward interventions like incorrect dicom informations need to be determined first. This is only possible with adequate quality control and assurance. If these are not recognized, significant effects on the data cannot be ruled out. Transferring this number of 56% interventions needed to preclinical systems would result in approximately 60,480 rodents potentially be affected by scanner miscalibration and/or other scanner intervention to reach acceptable quantitative parameters (SUVs) [65]. Understanding and incorporating QC/QA in preclinical experimental design improves the accuracy of scientific outcomes, the robustness of the results and keeps the welfare of the laboratory animals at the forefront, i.e. reducing pointless radiation exposure (radiotracer and ionizing radiation), anaesthesia, radiotracer or CT contrast injections and potentially reduces the number of animals required overall.

Additionally, the detailed reporting of procedures (including QC/QA validation), protocols and interventions carried out in a study, including the QC testing, according to the ARRIVE guidelines (https://arriveguidelines.org/arrive-guidelines) will strengthen the reproducibility and reliability of the acquired data and, hence proving scientific integrity [8].

## Data Availability

Not applicable.
